# Integrative Multi-Omics Analysis Identifies an *SPP1*-Associated Spatial Mesenchymal–Myeloid Program in Glioblastoma

**DOI:** 10.3390/genes17060610

**Published:** 2026-05-28

**Authors:** Ying Wang, Dong Zhou, Zhen Hong

**Affiliations:** 1 Department of Neurology, West China Hospital of Sichuan University, Chengdu 610041, China; yingwang12138@163.com (Y.W.); zhoudong66@yahoo.de (D.Z.); 2 Institute of Brain Science and Brain-Inspired Technology, West China Hospital, Sichuan University, Chengdu 610041, China

**Keywords:** *SPP1*, glioblastoma, multi-omics, myeloid cells, mesenchymal-like state

## Abstract

**Background**: Glioblastoma (GBM) is characterized by pronounced transcriptional plasticity and a highly structured immune microenvironment, yet the molecular features associated with tumor-state transitions and immune remodeling remain incompletely understood. **Methods**: We used an integrative multi-omics framework to examine how secreted phosphoprotein 1 (*SPP1*) relates to tumor microenvironment organization in human gliomas. **Results:** Single-cell analyses associated *SPP1* with myeloid populations, mesenchymal-like (MES-like) malignant states, inflammatory regulatory programs, and inferred ligand–receptor co-expression patterns involving *SPP1*–*CD44* and *SPP1*–integrin pairs. Spatial transcriptomic analyses showed that *SPP1*-high regions were enriched for estimated myeloid abundance, MES-like tumor signal, and ECM/angiogenic programs, supporting an *SPP1*-associated spatial mesenchymal–myeloid program in GBM. Computational perturbation analyses provided network-level support for *SPP1*–*CD44*-associated stress-responsive programs. HPA immunohistochemistry provided tissue-level protein context for SPP1 and related mesenchymal/receptor-associated components. Ivy GAP analysis showed enrichment of *SPP1*-associated features in core-like anatomic compartments, and CODEX spatial protein imaging provided antibody-panel-based contextual support for mesenchymal–myeloid-associated features. In the TCGA-GBM cohort, elevated *SPP1* expression and an *SPP1*-associated mesenchymal signature were associated with poorer overall survival. **Conclusions**: These findings support an inferential model in which *SPP1* is associated with spatial mesenchymal–myeloid organization in GBM and nominate *SPP1*-associated programs as candidate readouts of tumor plasticity, inflammatory myeloid remodeling, and spatial tumor microenvironment organization.

## 1. Introduction

Glioblastoma (GBM) remains the most aggressive primary malignancy of the central nervous system and is characterized by marked cellular heterogeneity, therapeutic resistance, and inevitable recurrence [1]. Despite advances in surgical resection, radiotherapy, and chemotherapy, the prognosis of patients with GBM remains poor [2]. A defining feature of GBM is its pronounced transcriptional plasticity, whereby malignant cells dynamically transition among multiple cellular states, including astrocyte-like (AC-like), oligodendrocyte progenitor-like (OPC-like), neural progenitor-like (NPC-like), and mesenchymal-like (MES-like) programs [1]. Among these, the MES-like state has been consistently associated with inflammatory signaling, hypoxia, treatment resistance, and adverse clinical outcome [3]. However, the molecular features linking mesenchymal transition to tumor microenvironmental remodeling remain incompletely understood.

The GBM microenvironment is dominated by tumor-associated macrophages and other myeloid cells, which may constitute 30–50% of the tumor mass. These populations play central roles in extracellular matrix remodeling, angiogenesis, immunoregulation, and tumor invasion [4]. Increasing evidence indicates that MES-like malignant cells and tumor-associated myeloid populations are closely coupled rather than operating as isolated compartments [3,5]. Inflammatory pathways, including NF-κB and JAK–STAT signaling, have been implicated in mesenchymal transition and immune remodeling, raising the possibility that shared molecular features may link tumor plasticity with microenvironmental change [6]. Nevertheless, how these compartments are spatially organized within intact tumor tissue, and which candidate molecular axes may be associated with their local coupling, remain unresolved.

Secreted phosphoprotein 1 (*SPP1*), also known as osteopontin, is a multifunctional extracellular matrix-associated cytokine involved in cell adhesion, migration, and immune regulation [7]. Elevated *SPP1* expression has been reported across multiple cancer types and has been associated with tumor progression, macrophage recruitment, and poor prognosis [8]. In glioma, *SPP1* is frequently enriched in myeloid populations [9], but its relationship to mesenchymal tumor states and its potential involvement in spatially organized tumor–immune programs remain unclear. Importantly, prior studies based on bulk transcriptomics do not provide sufficient resolution to identify the cellular sources of *SPP1* or to delineate how *SPP1*-associated programs are coordinated across distinct tumor and microenvironmental compartments. Recent advances in single-cell and spatial transcriptomics now provide an opportunity to address these questions at substantially higher resolution [10]. These approaches make it possible to define cellular states, infer regulatory and intercellular programs, and place these features within intact tissue architecture, thereby moving beyond descriptive profiling toward candidate organizational features of tumor microenvironment interactions.

In this study, we integrated single-cell and spatial transcriptomic analyses with inferred ligand–receptor co-expression analysis, computational perturbation analysis, and orthogonal tissue- and protein-level contextual datasets to examine how *SPP1* relates to mesenchymal tumor states, myeloid activation, and local tissue organization in GBM. Through this integrative multi-omics framework, we sought to determine whether *SPP1*-associated programs are linked to spatial mesenchymal–myeloid organization and immune remodeling in GBM.

## 2. Materials and Methods

### 2.1. Single-Cell RNA Sequencing Data Processing

Publicly available single-cell transcriptomic datasets were obtained from GSE182109 and GSE174554 [11,12]. Raw count matrices generated using the 10× Genomics platform were processed in R using Seurat (v5.2.1) [13]. Cells expressing fewer than 200 genes, more (10× Genomics, Pleasanton, CA, USA) than 7500 genes, or with mitochondrial gene content greater than 15% were excluded. These thresholds were selected to remove low-complexity cells, potential doublet-like high-feature cells, and cells with high mitochondrial content while retaining the broad transcriptional diversity of glioma samples. Data were log-normalized with a scale factor of 10,000, and 2000 highly variable genes were identified using the variance-stabilizing transformation method. Based on principal component analysis (PCA) diagnostic evaluation and the cumulative variance explained by the principal components, 30 PCs were retained for integration and visualization, whereas the shared nearest-neighbor graph for clustering used the first 20 PCs. The first 20 and 30 PCs explained approximately 89.1% and 94.0% of the cumulative PCA variance, respectively (Supplementary Table S1). Batch effects across samples were corrected using Harmony (v1.2.3) [14], and UMAP embeddings were generated based on Harmony-corrected components. Louvain clustering was performed at a resolution of 0.5. As a robustness check, clustering was repeated at resolutions 0.3, 0.5, and 0.8 using the RNA shared nearest-neighbor graph; resolution 0.5 balanced major-lineage purity with interpretability and showed high agreement with resolution 0.8 (Supplementary Figure S1). Potential doublets were identified using DoubletFinder (v2.0.4) [15], and only singlet cells were retained for downstream analyses.

Cell-type annotation was performed using established marker genes and was further evaluated using SingleR (v2.8.0) with reference datasets from the Human Primary Cell Atlas and Blueprint/ENCODE [16]. Malignant glioma-state annotations were assigned using canonical glioma-state markers and malignant-state gene programs, followed by re-clustering to resolve AC-like, OPC-like, NPC-like, and MES-like states. Myeloid subclusters were annotated using marker genes and are described as M1-like or M2-like only as transcriptional/marker-based shorthand rather than as fixed macrophage polarization states. Differential gene expression analysis was performed using the Wilcoxon rank-sum test with Benjamini–Hochberg correction where multiple features were tested. Gene program activity was quantified using AddModuleScore. Stemness and MES-like scores were calculated using curated glioma-state gene sets derived from prior glioma-state literature [1], whereas glycolysis, oxidative phosphorylation, hypoxia, and fatty acid oxidation scores were calculated using curated metabolic gene sets from msigdbr/MSigDB collections.

### 2.2. Transcriptional Regulatory Analysis

Regulatory module inference was performed using scRegClust (v0.2.2) based on scaled expression matrices [17]. Functional enrichment analysis of identified modules was conducted using clusterProfiler (v4.14.6) with Gene Ontology annotation [18].

### 2.3. Cell–Cell Communication Analysis

Cell–cell communication analysis was performed using CellChat (v1.6.1) with the human CellChatDB ligand–receptor resource [19]. Because the analysis estimates communication probability from group-level ligand and receptor expression, the results were interpreted as inferred ligand–receptor co-expression probabilities rather than direct evidence of functional signaling or physical interaction. The CellChatDB.human database was restricted to the Secreted Signaling category for the primary communication analysis. The CellChat object used for the primary communication analysis contained 16 major cell groups, 1280 CellChatDB ligand–receptor interactions, 1236 significant ligand–receptor interactions, a 16 × 16 × 1236 ligand–receptor probability array, and a 16 × 16 × 32 pathway-level probability array. CellChat communication probabilities were computed using triMean averaging with raw.use = TRUE, nboot = 100, and seed.use = 1. *SPP1* pathway interactions and pathway-level matrices were exported from the CellChat object to determine ligand–receptor contributions, source–target probabilities, and pathway-level summaries. To support reproducibility of the *SPP1*-focused analysis, we exported the *SPP1*-related nonzero source–target communication table from the CellChat workflow, including source and target cell groups, ligand–receptor pairs, communication probabilities, *p*-values, pathway annotation, and supporting evidence (Supplementary Table S2).

### 2.4. Spatial Transcriptomic Analysis

Spatial transcriptomic profiling was performed using the 10× Genomics Visium platform. Spatial data were processed in Seurat (v5.2.1). Each section was normalized using SCTransform and integrated using Harmony (v1.2.3) based on the principal component space. Clustering was conducted at a resolution of 0.5, and gene program activity scores were computed using AddModuleScore. Cell-type deconvolution was performed using RCTD implemented in spacexr (v2.2.1), with the single-cell dataset used as the reference [20]. Estimated cell-type proportions were incorporated into the spatial object metadata for downstream analysis.

To quantify the coordinated spatial organization of *SPP1* expression, estimated myeloid abundance, and MES-like tumor states, a composite *SPP1*–myeloid–MES score was generated for each spatial spot. Briefly, normalized *SPP1* expression, the estimated myeloid proportion from RCTD, and the MES-like signature score were each standardized by z-score transformation across spots within each section and then summed with equal weight. This equal-weight score was used as an exploratory visualization and integration metric, not as a validated biological index; the individual components were also examined separately. The MES-like signature score was calculated using the same MES-like gene set applied in the single-cell analyses. Ligand–receptor pairs were curated from the NicheNet human ligand–receptor network [21]. To estimate spot-level *SPP1*-associated ligand–receptor co-abundance, scores were calculated within each spot by multiplying *SPP1* expression by the expression of its cognate receptors (*CD44*, *ITGAV*, and *ITGB1*), and then summing the resulting products. These scores were interpreted as proxies for local ligand–receptor co-abundance rather than as direct measurements of cell–cell interaction or *SPP1* signaling activity.

Spatial adjacency relationships were modeled using k-nearest-neighbor graphs (k = 6), chosen to represent the immediate local neighborhood scale of Visium spots. Spots with the highest combined *SPP1*-associated co-abundance score, defined as the sum of *SPP1*–*CD44*, *SPP1*–*ITGAV*, and *SPP1*–*ITGB1* co-abundance scores, were identified as interaction-enriched hotspots for visualization. The top 5% hotspot threshold was used as a descriptive high-expression visualization cutoff rather than as a binary biological boundary. Robustness analyses compared top 5% and top 10% SPP1-high hotspot definitions, repeated local-neighborhood analyses using k = 4, 6, and 8 nearest neighbors, and reported continuous Spearman correlations alongside thresholded hotspot comparisons (Supplementary Tables S3 and S4; Supplementary Figures S2 and S3). Immediate neighboring spots were extracted to construct local microenvironmental subgraphs. Within these subgraphs, a co-abundance metric based on *SPP1* expression and cumulative receptor abundance was used to visualize regions with elevated *SPP1*-associated co-abundance. Associations between *SPP1*-related scores and MES-like tumor signal, estimated myeloid abundance, hypoxia, ECM, and angiogenesis signatures were evaluated using two-sided Wilcoxon rank-sum tests and Spearman correlation analyses. When multiple features were tested within the same analysis, *p* values were adjusted using the Benjamini–Hochberg method.

### 2.5. In Silico Computational Perturbation Analysis

To examine whether the proposed *SPP1*-associated mesenchymal–myeloid spatial program was compatible with perturbation-level network changes, we performed compartment-specific computational perturbation analysis using the annotated single-cell RNA-seq dataset. Based on the expression distribution of *SPP1* and *CD44* across cell populations, M2-like macrophage-associated cells were selected for *SPP1* perturbation, and MES-like malignant cells were selected for *CD44* perturbation. The myeloid subset used for computational perturbation analysis contained 23,126 cells, including 3903 M2-like macrophage-associated cells. Donor-balanced sampling was performed after applying a minimum target cell threshold of 25 cells per donor/sample. This design was intended to model perturbation of the *SPP1*-expressing myeloid compartment and the *CD44*-expressing MES-like malignant compartment, while remaining a computational network analysis rather than experimental perturbation validation. Computational perturbation analysis was performed using scTenifoldKnk [22] across five donor-balanced repeated runs. For the *SPP1* perturbation model, 430 M2-like macrophage-associated cells were sampled per repeat. For each repeat, cells were sampled by donor/sample, with up to 80 cells sampled per donor/sample. Donor/sample groups with fewer than 25 target cells were excluded from that perturbation model. Genes were filtered by expression frequency, ribosomal, mitochondrial, hemoglobin, and immunoglobulin genes were removed, and up to 2500 highly variable genes plus the target gene were retained. scTenifoldKnk was run with nc_nNet = 10, nc_nComp = 3, nc_q = 0.9, and td_K = 3. Perturbation results were summarized using the median perturbation Z-score (median Z), the fraction of repeats in which a gene was significant (stable fraction), and the minimum adjusted *p*-value across repeats (minimum *p*.adj). Top stable perturbed genes were visualized as ranked bar plots. To characterize pathway-level effects, the top 100 ranked perturbed genes from each model were subjected to GO Biological Process enrichment analysis. In parallel, Hallmark GSEA was performed using genes ranked by median perturbation Z-score after excluding the perturbed gene itself. Hallmark gene sets were obtained from msigdbr, and enrichment analysis was performed using clusterProfiler.

### 2.6. HPA Pathology/IHC Support and Ivy GAP Transcriptomic Anatomic-Context Analysis

To obtain tissue-level contextual support for components of the SPP1-associated mesenchymal–myeloid framework, we first examined immunohistochemistry (IHC) images from the Human Protein Atlas (HPA) pathology resource [23]. Representative GBM sections were reviewed for SPP1, CD44, ITGAV, ITGB1, and VIM. Image selection was qualitative and focused on representative tumor sections showing overall staining distribution and signal intensity. HPA images were interpreted as tissue-level context, not as experimental validation.

We next analyzed RNA-seq data from the Ivy Glioblastoma Atlas Project (Ivy GAP) to assess whether *SPP1*-related programs showed preferential enrichment across GBM anatomic compartments [24]. This dataset contains 122 RNA samples from 10 tumors spanning Leading Edge (LE), Infiltrating Tumor (IT), Cellular Tumor (CT), Microvascular Proliferation (MP), and Pseudopalisading Cells Around Necrosis (PCAN). Expression values were provided as normalized FPKM values. For the Ivy GAP analysis, gene-level expression was matched to sample-level structure annotations using the provided gene and sample metadata tables. *SPP1* expression was quantified as log2(FPKM + 1) and visualized at both the sample level and after tumor-level averaging to assess robustness across tumors. For compartment-level comparison, LE and IT were grouped as edge/infiltrative, whereas CT, MP, and PCAN were grouped as core-like.

To extend the analysis beyond a single gene, we calculated predefined module scores representing *SPP1*-associated, myeloid, mesenchymal, and hypoxia/vascular programs. The *SPP1*-associated module included *SPP1*, *CD44*, *ITGAV*, and *ITGB1*; the myeloid module included *CD163*, *APOE*, *HLA-DRA*, *AIF1*, *TYROBP*, *CSF1R*, and *ITGAM*; the mesenchymal module included *VIM*, *FN1*, and *CHI3L1*; and the hypoxia/vascular module included *SLC2A1*, *CA9*, *VEGFA*, and *PECAM1*. Module scores were calculated as the mean z-scored expression of the genes within each set and were compared across Ivy GAP structures and between edge/infiltrative and core-like compartments. We also generated a structure-level heatmap of selected genes spanning myeloid, mesenchymal/receptor, and hypoxia/vascular programs and examined the associations between *SPP1* expression and each module across anatomical samples.

### 2.7. Spatial Protein-Level Support for the SPP1-Associated Mesenchymal–Myeloid Features

To obtain orthogonal spatial protein-level contextual support for *SPP1*-associated mesenchymal–myeloid features inferred from the transcriptomic analysis, we analyzed CODEX data from the infiltrative region (INF) and T1-enhancing tumor region (T1) of the ZH916 sample using a single-cell spatial analysis framework adapted from Greenwald et al. [25]. Cell-level detection matrices were integrated with annotation files to generate a spatially resolved antibody-based protein map containing marker intensities, x–y coordinates, and cell-type labels for each segmented cell. Because the CODEX antibody panel did not directly measure the full *SPP1* transcriptional program, all CODEX-derived scores were interpreted as limited antibody-panel surrogate readouts rather than direct measurements of SPP1 signaling.

For regional composition analysis, the relative abundance of annotated cell populations in the INF and T1 regions was calculated and visualized as stacked bar plots. To examine spatial organization, cells were projected back to their original tissue coordinates and displayed as cell-type-colored scatter plots. To identify myeloid-enriched spatial domains, cells annotated as myeloid lineage cells were subsetted and analyzed separately in INF and T1 using two-dimensional kernel density estimation, and the resulting density contours were visualized as regional myeloid density maps.

To characterize regional- and cell-type-associated protein features, normalized marker intensities were summarized across annotated cell populations and regions and displayed as a heatmap. In addition, three proxy scores were calculated at the single-cell level: an SPP1-like myeloid proxy (CD163, APOE, CHI3L1, CD11c, HLA-DR, and CD14), a mesenchymal-compatible proxy (CD44, VIM, FN1, CHI3L1, and GLUT1), and a CD44 receptor-side proxy (CD44). For proxies defined by multiple markers, scores were calculated as the mean of z-scored expression values across the corresponding antibody channels; the CD44 receptor-side proxy was represented by the z-scored CD44 channel alone. These scores were then compared across annotated cell types and between the INF and T1 regions using violin plots.

### 2.8. Clinical and Survival Analysis

Bulk RNA sequencing and clinical data were obtained from The Cancer Genome Atlas (TCGA), including the TCGA-LGG and TCGA-GBM glioma cohorts. Overall survival was defined as the interval from diagnosis to death or last follow-up. For GBM-focused prognosis analyses, GBM samples were analyzed separately. Patients were stratified according to median *SPP1* expression or median *SPP1*-associated mesenchymal signature score. The median split was selected as a simple pre-specified exploratory dichotomization rather than as an optimized clinical cutoff. Kaplan–Meier survival curves were compared using the log-rank test. The *SPP1*-associated mesenchymal signature comprised *SPP1*, *CD44*, *VIM*, *CHI3L1*, and *FN1*, and sample-level signature scores were calculated using GSVA (method = ‘ssgsea’) [26]. Multivariable Cox proportional hazards analysis was performed in GBM cases with available age, sex, IDH status, 1p/19q codeletion status, and MGMT status, with *SPP1* or signature group included together with these variables as covariates. Hazard ratios with 95% confidence intervals were reported. Time-dependent receiver operating characteristic analysis was performed to assess internal cohort discriminative performance at 1-, 2-, and 3-year survival endpoints.

For molecular co-expression and immune signature analyses across glioma cohorts, TCGA-LGG and TCGA-GBM samples were analyzed as a combined glioma cohort. These analyses were interpreted as associations across glioma cohorts and were considered potentially influenced in part by underlying grade structure. Immune cell infiltration was estimated using xCell (v1.1.0), and associations between the *SPP1*-associated mesenchymal signature score and immune cell infiltration were evaluated using Spearman correlation [27].

### 2.9. Statistical Analysis

All statistical analyses were performed in R software (R Foundation for Statistical Computing, Vienna, Austria). Statistical tests were two-sided unless otherwise specified. *p*-values were adjusted using the Benjamini–Hochberg method when multiple features or pathways were tested; for single planned comparisons, unadjusted two-sided *p*-values were reported. An adjusted *p*-value of less than 0.05 was considered statistically significant. The major R packages and computational tools used in this study included Seurat, Harmony, DoubletFinder, SingleR, scRegClust, clusterProfiler, CellChat, spacexr/RCTD, NicheNet, scTenifoldKnk, msigdbr/MSigDB, GSVA, and xCell, with versions reported in the corresponding Methods sections where available.

### 2.10. Network–Resource Transparency and Reproducibility

The network-related analyses in this study used several distinct resources and graph definitions rather than a single generic protein–protein interactome. CellChat analyses used CellChatDB.human ligand–receptor pairs from the Secreted Signaling subset and generated group-level CellChat probability networks; the CellChat object contained pathway-level *SPP1* probabilities and was used to export *SPP1* ligand–receptor contributions, source–target probability matrices, and pathway-level summaries. *SPP1*-related CellChat outputs and session information were exported from the CellChat workflow as described in Section 2.3. Spatial ligand–receptor analyses used the NicheNet human ligand–receptor network to focus on *SPP1*–*CD44*, *SPP1*–*ITGAV*, and *SPP1*–*ITGB1* co-abundance. Spatial neighborhood graphs were k-nearest-neighbor spot graphs; the main analysis used k = 6 and robustness analyses repeated the local-neighborhood calculations at k = 4, 6, and 8. scTenifoldKnk generated inferred gene regulatory networks within donor-balanced cell subsets, and perturbation effects were ranked by median Z, stable fraction, and adjusted *p*-values across repeated runs.

Because the network analyses were based on ligand–receptor resources, spatial neighborhood graphs, and inferred single-cell regulatory networks rather than a confidence-thresholded global protein–protein interaction interactome, STRING-, BioGRID-, or IntAct-specific parameters such as global PPI confidence scores, source channel filters, and global node degree statistics were not applicable.

## 3. Results

### 3.1. Human Glioma Single-Cell and Spatial Transcriptomic Datasets

For single-cell analyses, we collected publicly available single-cell transcriptomic datasets from GSE182109 and GSE174554, comprising 15 GBM patient samples [11,12]. For spatial transcriptomic analyses, we integrated six glioma samples, including two oligodendrogliomas, two astrocytomas, and two glioblastomas, from GSE237183 and a previously published spatial transcriptomic dataset [25,28]. This cohort enabled cross-grade comparison of spatial tumor architecture while retaining a primary focus on GBM-associated mesenchymal–myeloid organization.

### 3.2. SPP1 Expression in Myeloid Cells and MES-like Tumor States

**Figure 1:**
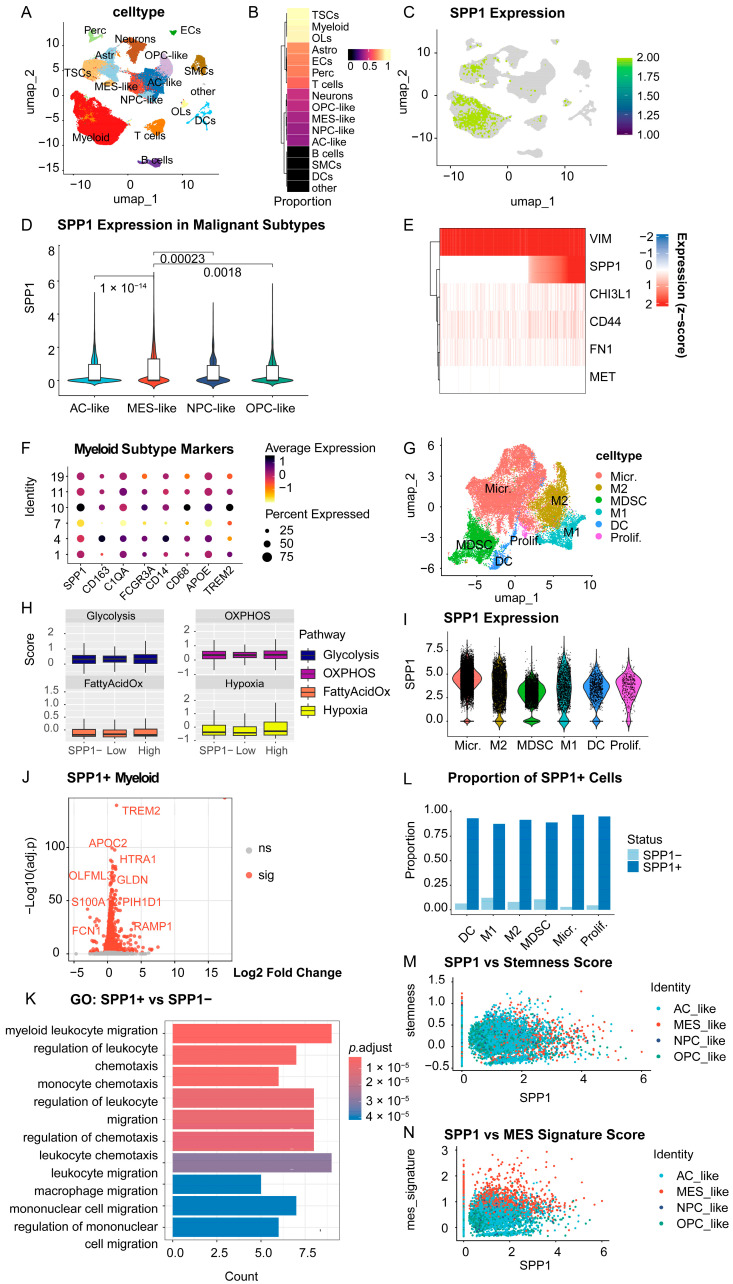
Cellular distribution and transcriptional features of *SPP1* in GBM. (**A**) UMAP projection of integrated single-cell transcriptomic data from GBM samples, colored by annotated malignant states and major non-malignant cell populations, including mesenchymal-like (MES-like), astrocyte-like (AC-like), oligodendrocyte progenitor-like (OPC-like), neural progenitor-like (NPC-like), myeloid cells (Myeloid), dendritic cells (DCs), endothelial cells (ECs), smooth muscle cells (SMCs), and pericytes (Perc). (**B**) Heatmap showing the proportion of *SPP1*^+^ cells across major cell populations in GBM. (**C**) UMAP feature plot showing the spatial distribution of *SPP1* expression across all cell populations. (**D**) Violin plots displaying *SPP1* expression levels across malignant glioma cell states. (**E**) Heatmap showing Z-score-normalized expression levels of *SPP1* and canonical mesenchymal markers (*VIM*, *CHI3L1*, *CD44*, *FN1*, and *MET*) in malignant cells, ordered by *SPP1* expression level. (**F**) Dot plot illustrating average expression levels and the percentage of cells expressing key myeloid markers across myeloid subpopulations. (**G**) UMAP projection of myeloid cells with clusters annotated based on canonical marker gene expression: Micr. (microglia), M2 (M2-like macrophage-associated cells), MDSC (myeloid-derived suppressor cells), M1 (M1-like macrophage-associated cells), DC (dendritic cells), and Prolif. (proliferating myeloid cells). These labels denote marker-based macrophage-associated transcriptional states rather than fixed macrophage polarization categories. (**H**) Box plots showing pathway enrichment scores for key metabolic gene sets—glycolysis, oxidative phosphorylation (OXPHOS), fatty acid oxidation (FattyAcidOx), and hypoxia—in malignant glioma cells stratified by *SPP1* expression level (*SPP1*^−^, *SPP1*^+^ low, and *SPP1*^+^ high). (**I**) Violin plots showing *SPP1* expression levels across distinct myeloid subsets. (**J**) Volcano plot showing differentially expressed genes (DEGs) between *SPP1*^+^ and *SPP1*^−^ myeloid cells. Genes meeting significance criteria (|log_2_ fold change| > 1 and adjusted *p*-value < 0.05) are highlighted in red; non-significant genes are shown in gray. (**K**) Gene Ontology (GO) enrichment analysis of DEGs between *SPP1*^+^ and *SPP1*^−^ myeloid cells. Bar plot showing the top significantly enriched GO terms, including leukocyte migration and chemotaxis. (**L**) Bar plot showing the proportion of *SPP1*^+^ cells within each myeloid subset. (**M**,**N**) Scatter plots showing the distribution of *SPP1* expression relative to stemness (**M**) and mesenchymal signature scores (**N**) across malignant glioma states.

After stringent quality control, 70,036 high-quality cells were retained for downstream analysis. Unsupervised Louvain clustering identified transcriptionally distinct populations across tumors. Major malignant and non-malignant compartments were assigned using canonical marker genes, glioma-state signatures, and SingleR-supported annotation. Malignant cells segregated into transcriptional states consistent with established glioma programs, including AC-like, OPC-like, NPC-like, and MES-like states (Figure 1A). The non-malignant compartment was composed mainly of myeloid cells together with lymphoid populations. Given prior evidence linking TAMs to *SPP1* expression in glioma, we further subclustered myeloid populations (clusters 1, 4, 7, 10, 11, 19), identifying microglia, M1-like and M2-like macrophage-associated states, dendritic cells (DCs), myeloid-derived suppressor cells (MDSCs), and proliferating myeloid subsets (Figure 1F,G).

Across the single-cell dataset, *SPP1* expression was primarily detected to the myeloid cell cluster, as shown by the feature plot and cell-type distribution summaries (Figure 1B,C). Quantitative comparisons demonstrated that *SPP1* expression was higher in myeloid populations than in malignant subtypes (Figure 1D,I,L), supporting myeloid populations as the principal cellular source of *SPP1* in this dataset.

Within malignant cells, *SPP1* expression was preferentially enriched in MES-like states. Heatmap analysis showed coordinated expression of *SPP1* with mesenchymal markers, including *VIM* and other canonical MES-associated genes (Figure 1E). Consistently, module scoring showed that malignant cells with elevated *SPP1* expression were more frequently observed among cells with higher MES-like signature scores (Figure 1N). In contrast, the relationship between *SPP1* expression and stemness-related module scores was less consistent across malignant populations (Figure 1M). Although *SPP1* expression was detectable across multiple malignant subtypes, MES-like cells exhibited a broader and more prominent expression range than other transcriptional states. Metabolic pathway analysis further indicated that malignant cells with higher *SPP1* expression exhibited increased hypoxia, fatty acid oxidation, oxidative phosphorylation, and glycolysis signatures (Figure 1H), suggesting that *SPP1* expression is associated with metabolically adaptive and stress-related tumor states.

In the myeloid compartment, differential expression analysis comparing *SPP1*-high and *SPP1*-low cells identified genes enriched in migration, inflammatory response, and extracellular matrix remodeling pathways (Figure 1J,K), consistent with an activated myeloid-associated transcriptional phenotype.

### 3.3. Transcription Factors Associated with SPP1 Regulatory Programs

**Figure 2:**
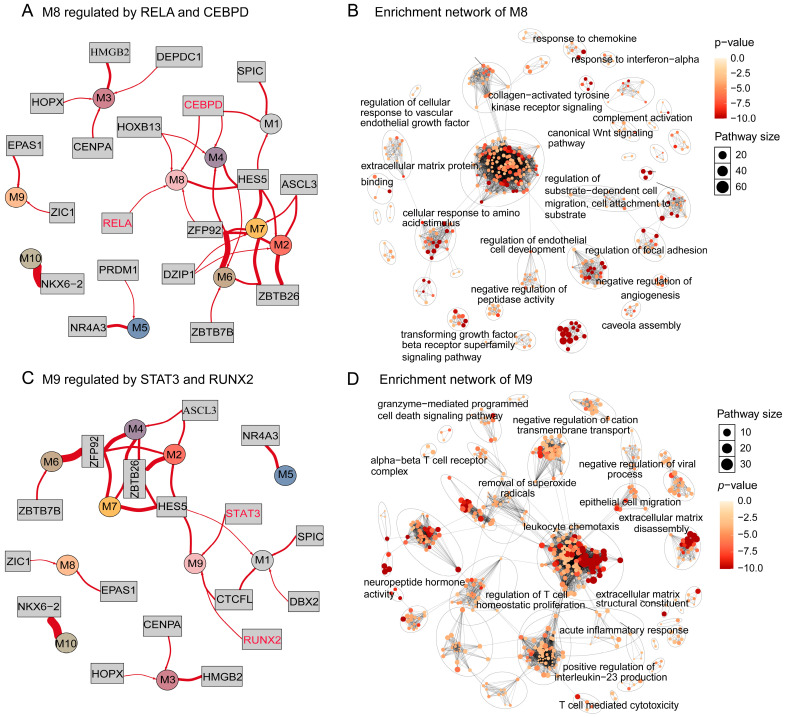
Transcriptional regulatory modules linked to *SPP1*-related programs. (**A**,**C**) Transcriptional regulatory networks inferred from single-cell data, showing co-regulated transcription factors (TFs) and their associated modules. *RELA*, *CEBPD*, *RUNX2*, and *STAT3* are transcription factors previously implicated in *SPP1*-associated inflammatory programs. Module 8 (M8) is co-regulated by *RELA* and *CEBPD* (**A**), whereas Module 9 (M9) is co-regulated by *RUNX2* and *STAT3* (**C**). Red arrows indicate inferred regulatory relationships between TFs and module genes. (**B**,**D**) GO enrichment networks for M8 and M9. M8 is enriched for extracellular matrix remodeling, VEGF signaling, canonical Wnt signaling, complement activation, and vascular development (**B**). M9 is enriched for immune-related pathways, including T cell-mediated cytotoxicity, leukocyte chemotaxis, interleukin production, and acute inflammatory response (**D**).

To investigate the transcriptional context associated with *SPP1*, we applied scRegClust to infer gene regulatory modules from the single-cell data. This analysis identified multiple co-regulated modules enriched for inflammatory and extracellular matrix-related pathways, suggesting coordinated transcriptional programs spanning malignant and myeloid compartments. Transcription factors previously implicated in inflammatory programs associated with *SPP1*, including *RELA*, *CEBPD*, *RUNX2*, and *STAT3*, were prominently represented in these inferred modules. Module 8, co-regulated by *RELA* and *CEBPD*, was enriched for pathways related to extracellular matrix remodeling, substrate-dependent cell migration, response to VEGF, canonical Wnt signaling, complement activation, and vasculature development, suggesting links between *SPP1*-associated programs and vascular and immune remodeling (Figure 2A,B). Module 9, co-regulated by *RUNX2* and *STAT3*, was enriched for immune activation pathways, including interleukin-23 production, T cell-mediated cytotoxicity, leukocyte chemotaxis, and acute inflammatory response (Figure 2C,D).

### 3.4. Inferred SPP1-Associated Ligand–Receptor Co-Expression Involving MES-like Malignant and Myeloid Compartments

**Figure 3:**
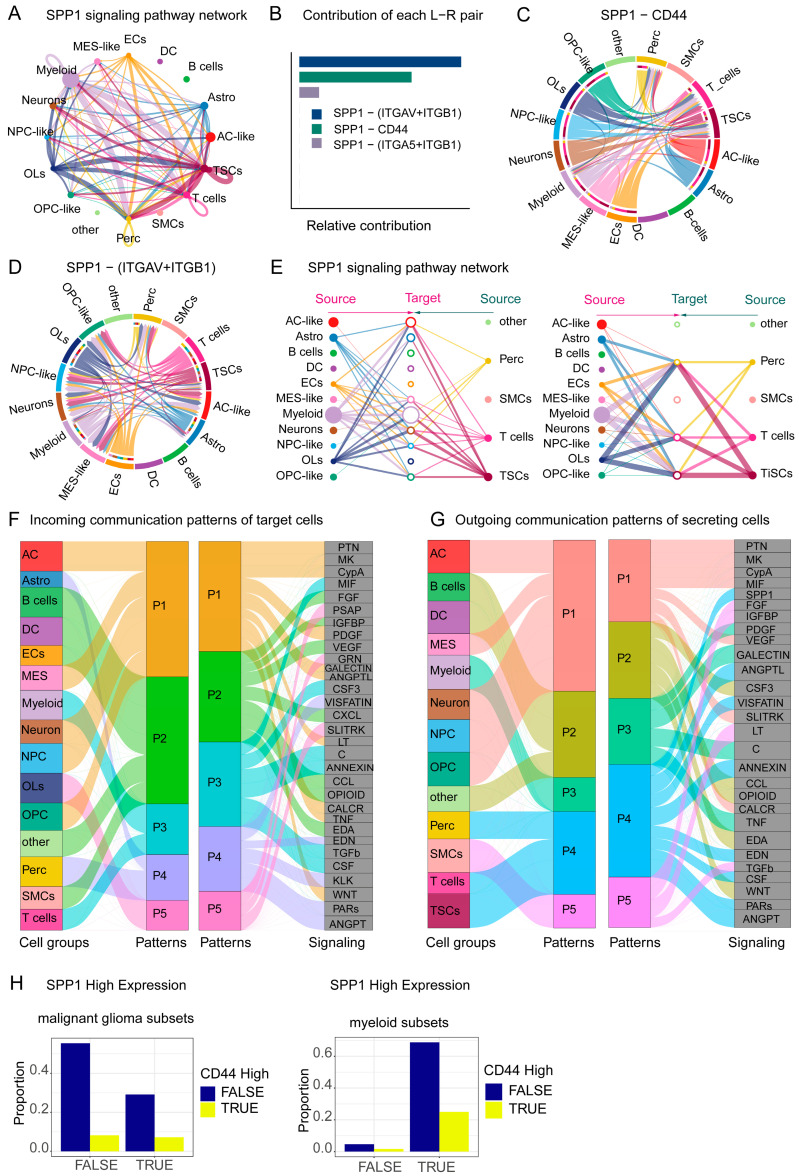
Inferred *SPP1*-associated ligand–receptor co-expression involving MES-like malignant cells and myeloid populations. (**A**) Circular plot showing the CellChat-inferred *SPP1* ligand–receptor probability network among major cell types. (**B**) Relative contributions of individual ligand–receptor pairs within the inferred *SPP1* pathway. Bar plot illustrating the proportional contribution of *SPP1*–*ITGAV*+*ITGB1*), *SPP1*–*CD44*, and *SPP1*–*ITGA5*+*ITGB1*) interactions to the total *SPP1* pathway probability. (**C**,**D**) Chord diagrams showing CellChat-inferred ligand–receptor co-expression probabilities involving MES-like malignant cells and myeloid populations for *SPP1*–*CD44* (**C**) and *SPP1*–(*ITGAV*+*ITGB1*) (**D**). These plots do not establish functional signaling or directionality. (**E**) Aggregated visualization of the inferred *SPP1* ligand–receptor probability network, showing MES-like malignant cells and myeloid populations in inferred ligand-expressing source and receptor-expressing receiver positions within the CellChat framework. (**F**,**G**) Sankey diagrams summarizing dominant CellChat-inferred outgoing and incoming ligand–receptor patterns across cell populations. (**H**) Co-expression analysis of *SPP1* and *CD44* in malignant glioma cells and myeloid subsets.

To investigate whether the inferred inflammatory regulatory context identified above was accompanied by coordinated ligand–receptor co-expression among annotated cell populations, we examined the *SPP1* pathway within the CellChat analysis (Supplementary Table S2). The inferred *SPP1*-associated ligand–receptor probability network showed broad co-expression patterns across malignant, stromal, and immune compartments (Figure 3A). Among *SPP1* ligand–receptor interactions, *SPP1*–(*ITGAV*+*ITGB1*), *SPP1*–*CD44*, and *SPP1*–(*ITGA5*+*ITGB1*) accounted for 58.0%, 35.6%, and 6.3% of total SPP1 ligand–receptor probability, respectively (Figure 3B). Chord diagrams showed inferred *SPP1*–*CD44* ligand–receptor co-expression probabilities involving MES-like malignant cells and myeloid populations (Figure 3C), whereas *SPP1*–(*ITGAV*+*ITGB1*) showed inferred ligand–receptor co-expression probabilities involving myeloid populations and MES-like malignant cells (Figure 3D). Co-expression analysis further showed overlapping *SPP1* and *CD44* expression in both malignant and myeloid subsets (Figure 3H). Aggregated network visualization further indicated that MES-like malignant cells and myeloid populations appeared in both inferred source and receiver positions within the CellChat *SPP1*-associated ligand–receptor probability network (Figure 3E). These source–receiver assignments should be interpreted as computationally inferred ligand–receptor co-expression probabilities rather than direct evidence of functional signaling or directionality.

To further delineate broader CellChat-inferred communication patterns within the glioma microenvironment, we decomposed inferred incoming and outgoing ligand–receptor patterns into five major patterns (Figure 3F,G). MES-like malignant cells were mainly associated with incoming pattern 1, which included ligands such as *PTN*, *PDGF*, and *WNT*, whereas myeloid populations were mainly associated with incoming pattern 3, which included *MIF*, *CSF3*, *TGF-β*, and complement ligands. For outgoing patterns, MES-like malignant cells were mainly associated with pattern 1, including *PTN*, *MIF*, and *VEGF*, whereas myeloid populations were mainly associated with pattern 3, which encompassed *PDGF*, *TNF*, and complement ligands.

### 3.5. Spatial Analyses Support an SPP1-Associated Mesenchymal–Myeloid Spatial Program in GBM

**Figure 4:**
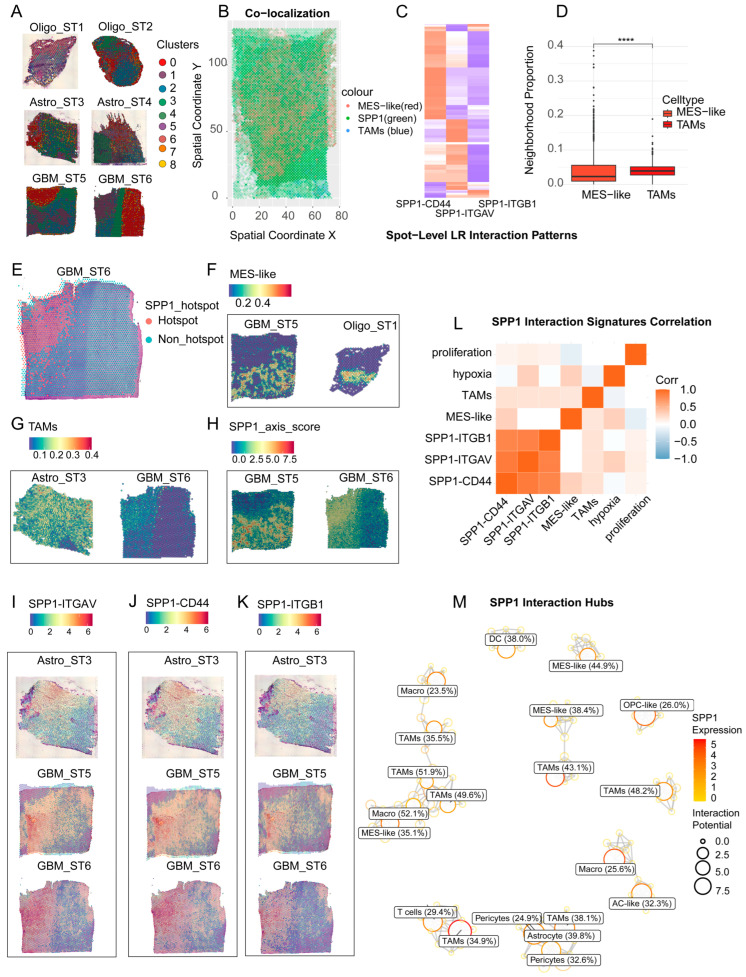
Spatial analyses support an *SPP1*-associated mesenchymal–myeloid spatial program in GBM. (**A**) Spatial distribution of transcriptionally defined spatial clusters across glioma tissues of three pathological grades. Spots are colored according to cluster annotation. (**B**) Spatial co-localization of *SPP1* expression with MES-like tumor signal and estimated myeloid abundance. Composite spatial map showing the distribution of MES-like tumor signal (red), *SPP1* expression (green), and estimated myeloid abundance (blue) within a representative glioma section. (**C**) Spot-level spatial co-abundance patterns of *SPP1* with its receptors. Heatmap showing Z-score-normalized spatial co-abundance scores between *SPP1* and *CD44*, *ITGAV*, and *ITGB1* across individual spots. Red indicates higher co-abundance intensity and blue indicates lower co-abundance intensity. (**D**) Neighborhood composition of *SPP1*-high spots. Boxplots showing the proportion of MES-like tumor signal and estimated myeloid abundance within the six nearest neighboring spots surrounding *SPP1*-high regions. Statistical significance was assessed using a two-sided Wilcoxon rank-sum test. **** *p* < 0.0001. Threshold and neighborhood size robustness analyses are provided in Supplementary Figures S2 and S3. (**E**) Spatial mapping of *SPP1* expression hotspots. Spatial plot showing regions corresponding to the top 5% of SPP1 expression values in a representative glioblastoma sample (GBM_ST6). The top 5% threshold was used for hotspot visualization rather than as a biological cutoff. (**F**,**G**) Spatial heatmaps showing MES-like tumor signal and estimated myeloid abundance across representative glioma samples. (**H**) Spatial distribution of a composite *SPP1*–myeloid–MES score in representative glioma tissues. Heatmap showing the combined score integrating *SPP1* expression, estimated myeloid abundance, and MES-like signature scores across representative samples. (**I**–**K**) Spatial co-abundance patterns of *SPP1* with individual receptors. Heatmaps showing *SPP1*–*ITGAV* (**I**), *SPP1*–*CD44* (**J**), and *SPP1*–*ITGB1* (**K**) co-abundance scores across multiple glioma samples (Astro_ST3, GBM_ST5, GBM_ST6). Higher scores indicate regions with elevated *SPP1*–receptor co-abundance. (**L**) Association between spatial *SPP1*–receptor co-abundance scores and biological programs. Heatmap displaying Spearman correlation coefficients between *SPP1*–*CD44*, *SPP1*–*ITGAV*, and *SPP1*–*ITGB1* co-abundance scores and spatial signatures of proliferation, hypoxia, estimated myeloid abundance, and MES-like tumor states across tissue spots. (**M**) Locally connected regions of elevated *SPP1*-associated co-abundance. Spatial graph visualization highlighting the top 20 spots with the highest combined *SPP1*–receptor co-abundance scores (orange nodes) and their first-order neighboring spots. Node size is proportional to the combined score, and node color reflects *SPP1* expression level. Labels indicate the deconvolved dominant cell type within each spot, defined as the cell type with the highest estimated proportion; numbers in parentheses represent the estimated proportion of that cell type.

To map *SPP1*-associated programs in situ, we deconvolved spatial transcriptomic datasets using RCTD and estimated cell-type proportions for each spatial spot. Spatial clustering revealed heterogeneous architectural domains across tumor grades (Figure 4A). In GBM samples, *SPP1*-high regions, defined as the top 5% of spots by *SPP1* expression for visualization, formed discrete spatial hotspots, such as spatial cluster 5 in GBM_ST6 (Figure 4E). Robustness analyses using both top 5% and top 10% hotspot definitions showed that *SPP1*-high GBM regions were enriched for estimated myeloid abundance, MES-like tumor signal, and ECM/angiogenic programs, consistent with a spatially restricted *SPP1*-associated mesenchymal–myeloid program in GBM (Supplementary Figure S2A). Although the relative contribution of these features varied across sections, the overall pattern indicated that *SPP1*-high regions preferentially localized to myeloid-rich and mesenchymal-associated tissue contexts with accompanying ECM/angiogenic features. Continuous Spearman analyses further supported this interpretation by showing positive associations between *SPP1* expression and these spatial features (Supplementary Figure S2B). Overlay of MES-like tumor signal and estimated myeloid abundance across representative samples showed spatially overlapping enrichment patterns in *SPP1*-high regions (Figure 4F,G). Triple-color visualization of *SPP1*-high spots, MES-like tumor signal, and estimated myeloid abundance showed substantial overlap, consistent with spatially restricted regions showing tumor–myeloid co-enrichment (Figure 4B). Spatial neighborhood analysis further supported enrichment of MES-like tumor signal and estimated myeloid abundance in the immediate vicinity of *SPP1*-high spots (Figure 4D).

To integrate these spatial features, we constructed an exploratory composite *SPP1*–myeloid–MES score by combining normalized *SPP1* expression with estimated myeloid abundance and MES-like tumor signal. High-scoring regions formed coherent spatial domains across representative glioma samples (Figure 4H), consistent with spatially restricted co-enrichment of *SPP1*-associated myeloid and mesenchymal features. Together with the hotspot- and correlation-based analyses above, this composite spatial pattern further supports an *SPP1*-associated mesenchymal–myeloid program in GBM.

We next calculated spot-level *SPP1*–*CD44*, *SPP1*–*ITGAV*, and *SPP1*–*ITGB1* co-abundance scores as proxies for local ligand–receptor co-abundance. These spatial maps showed structured regional enrichment patterns (Figure 4I–K), and a spot-level heatmap highlighted coordinated elevation of these scores across samples (Figure 4C). Spatial k-nearest-neighbor robustness analyses further showed that local enrichment patterns around *SPP1*-high regions were generally preserved across k = 4, 6, and 8, supporting the robustness of the inferred *SPP1*-associated spatial organization (Supplementary Figure S3). Correlation analyses showed that *SPP1*–*CD44* co-abundance scores were positively associated with MES-like tumor signal and estimated myeloid abundance, whereas *SPP1*–*ITGAV* and *SPP1*–*ITGB1* scores were associated with hypoxia and proliferation signatures (Figure 4L). Graph-based analysis of the top 20 co-abundance-enriched spots identified locally connected subgraphs enriched for spots with relatively higher *SPP1*-associated co-abundance (Figure 4M), suggesting a locally connected pattern of spatially concentrated *SPP1*-associated co-abundance.

### 3.6. Computational Perturbation Analysis Supports SPP1–CD44-Associated Stress-Responsive Programs

**Figure 5:**
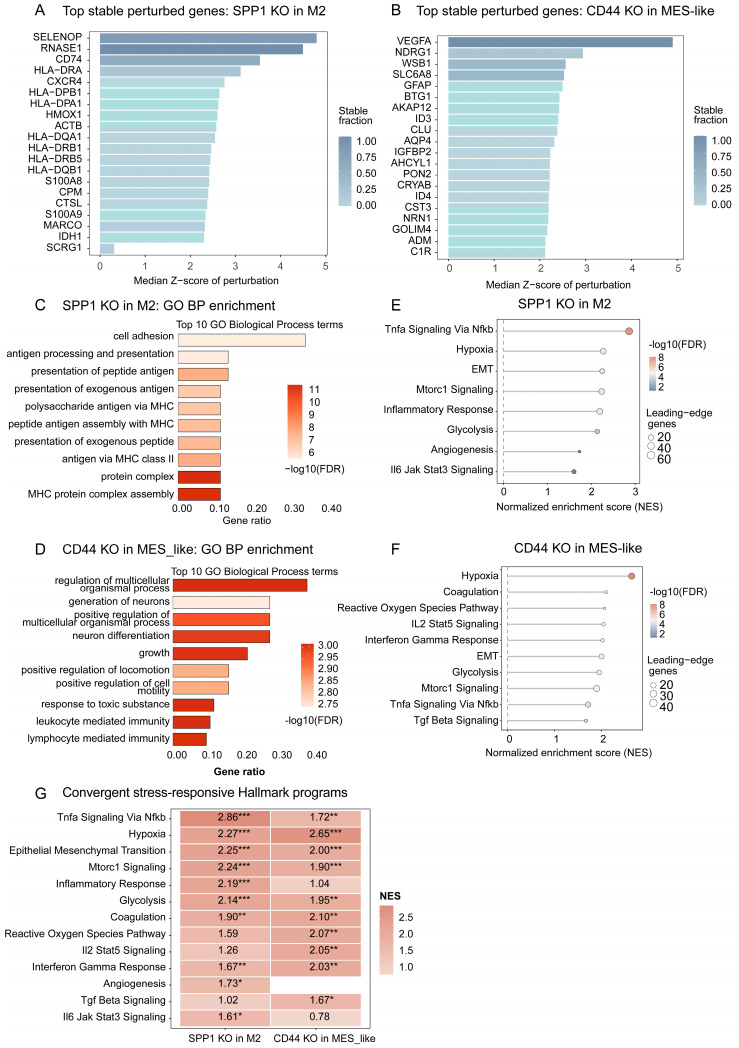
Computational perturbation analysis supports *SPP1*–*CD44*-associated stress-responsive network programs. (**A**) Top stable perturbed genes after computational perturbation of *SPP1* in M2-like macrophage-associated cells. Genes are ranked by median perturbation Z-score. Bar length indicates median perturbation Z-score, and bar color indicates stable fraction across repeated runs. (**B**) Top stable perturbed genes after computational perturbation of *CD44* in MES-like malignant cells. (**C**) GO enrichment analysis of top-ranked perturbed genes after *SPP1* perturbation in M2-like macrophage-associated cells. Bars indicate gene ratio and color indicates enrichment significance. (**D**) GO enrichment analysis of top-ranked perturbed genes after *CD44* perturbation in MES-like malignant cells. (**E**) Hallmark GSEA after computational *SPP1* perturbation in M2-like macrophage-associated cells. The x-axis indicates normalized enrichment score (NES), point size indicates leading-edge gene count, and color indicates enrichment significance. (**F**) Hallmark GSEA after computational *CD44* perturbation in MES-like malignant cells. (**G**) Integrated heatmap of selected Hallmark pathways across the two computational perturbation models. Tile color and overlaid values indicate NES. Shared enrichment patterns included TNFα/NF-κB signaling, hypoxia, epithelial–mesenchymal transition (EMT), mTORC1 signaling, and glycolysis. Significance annotations indicate Benjamini–Hochberg-adjusted *p*-values: * *p* < 0.05, ** *p* < 0.01, and *** *p* < 0.001.

Computational perturbation of *SPP1* in M2-like macrophage-associated cells revealed recurrent network-level perturbation patterns across five repeated donor-balanced runs (Figure 5A). As expected, the perturbed gene *SPP1* showed a strong perturbation signal; additional stable genes included *RNASE1* (stable fraction = 1.0, median Z = 4.49), *SELENOP* (stable fraction = 0.8, median Z = 4.80), and *CD74* (stable fraction = 0.6, median Z = 3.54). GO enrichment was dominated by cell adhesion and antigen processing/presentation programs (Figure 5C), suggesting preferential network-level changes in adhesion- and immune-presentation-related myeloid programs. Hallmark analysis further highlighted inflammatory and stress-responsive programs, including TNFα/NF-κB signaling, hypoxia, epithelial–mesenchymal transition, mTORC1 signaling, and glycolysis (Figure 5E).

Computational perturbation of *CD44* in MES-like malignant cells yielded a distinct but partially convergent perturbation profile (Figure 5B). GO enrichment highlighted programs related to cell motility, locomotion, growth, and stress response (Figure 5D). Hallmark analysis further showed enrichment of hypoxia-, EMT-, glycolysis-, inflammation-, and TGF-β-related programs, consistent with a stress-responsive and mesenchymal-associated perturbation profile (Figure 5F).

Comparison of Hallmark enrichment across the two computational perturbation models showed partial convergence on stress-responsive and mesenchymal-associated programs, including TNFα/NF-κB signaling, hypoxia, epithelial–mesenchymal transition, glycolysis, and mTORC1 signaling (Figure 5G). The myeloid *SPP1* perturbation was more prominently associated with inflammatory and angiogenic programs, whereas the MES-like *CD44* perturbation was more prominently associated with reactive oxygen species-, interferon-, and TGF-β-related programs.

### 3.7. HPA and Ivy GAP Provide Tissue-Level and Anatomic-Context Support for SPP1-Associated Programs in GBM

**Figure 6:**
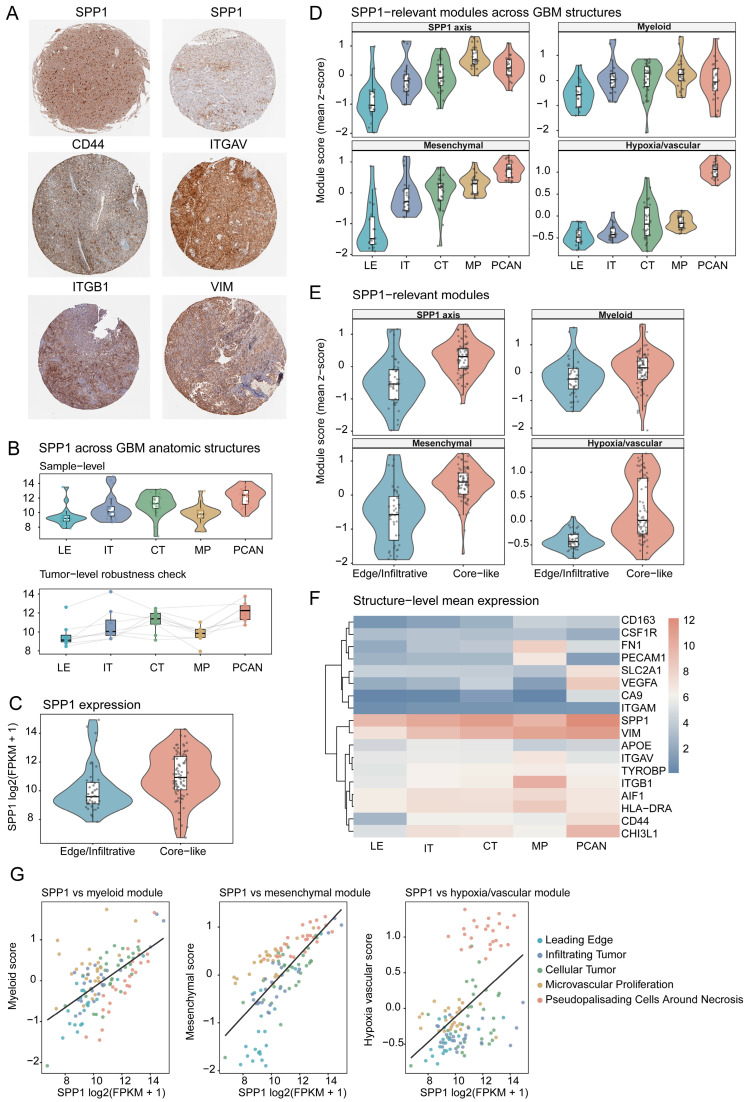
HPA and Ivy GAP provide tissue-level and anatomic-context support for *SPP1*-associated programs in GBM. (**A**) Representative Human Protein Atlas (HPA) immunohistochemistry images from GBM samples showing detectable staining for SPP1, CD44, ITGAV, ITGB1, and VIM. Two representative SPP1 images are shown. (**B**) *SPP1* expression across Ivy GAP anatomic structures. Upper panel, sample-level expression shown as log2(FPKM + 1) across Leading Edge (LE), Infiltrating Tumor (IT), Cellular Tumor (CT), Microvascular Proliferation (MP), and Pseudopalisading Cells Around Necrosis (PCAN). Lower panel, tumor-level mean expression showing a similar structural pattern after averaging within tumors. (**C**) Comparison of *SPP1* expression between edge/infiltrative (LE + IT) and core-like (CT + MP + PCAN) compartments. (**D**) Distribution of *SPP1*-associated, myeloid, mesenchymal, and hypoxia/vascular module scores across Ivy GAP structures. (**E**) Comparison of the same four modules between edge/infiltrative and core-like compartments. (**F**) Structure-level heatmap showing mean expression of selected genes representing myeloid, mesenchymal/receptor, and hypoxia/vascular programs across LE, IT, CT, MP, and PCAN. (**G**) Scatterplots showing *SPP1* expression plotted against the myeloid, mesenchymal, and hypoxia/vascular module scores across Ivy GAP samples.

Representative immunohistochemistry images from HPA GBM samples showed detectable staining for SPP1, CD44, ITGAV, ITGB1, and VIM in tumor sections (Figure 6A), providing tissue-level protein context for the presence of SPP1 and selected mesenchymal/receptor-associated components in GBM tissue.

Transcriptomic analysis of the Ivy Glioblastoma Atlas showed that *SPP1* was not uniformly distributed across annotated GBM anatomic structures. At the sample level, *SPP1* expression was lowest in Leading Edge, intermediate in Infiltrating Tumor and Microvascular Proliferation, higher in Cellular Tumor, and highest in Pseudopalisading Cells Around Necrosis (Figure 6B, upper). A similar pattern was observed after tumor-level averaging (Figure 6B, lower). When grouped into broader compartments, *SPP1* was relatively higher in core-like than in edge/infiltrative regions (Figure 6C).

This core-biased pattern extended to broader biological programs. Across Ivy GAP structures, the *SPP1*-associated, myeloid, mesenchymal, and hypoxia/vascular modules were all relatively enriched in CT/MP/PCAN, with particularly strong hypoxia/vascular enrichment in PCAN (Figure 6D). The same overall pattern persisted in the edge/infiltrative versus core-like comparison, with all four modules higher in the core-like compartment (Figure 6E). These findings support an anatomic-context association between *SPP1* expression and broader myeloid, mesenchymal, and hypoxia/vascular programs in GBM.

Structure-level mean-expression analysis further supported this pattern (Figure 6F). Genes representing myeloid, mesenchymal/receptor, and hypoxia/vascular programs were relatively enriched in CT/MP/PCAN. Consistently, *SPP1* expression showed concordant increases with the myeloid, mesenchymal, and hypoxia/vascular module scores across Ivy GAP samples (Figure 6G), consistent with coordinated transcriptomic variation across these programs.

### 3.8. CODEX Spatial Protein Imaging Provides Orthogonal Spatial Protein-Level Support for SPP1-Associated Mesenchymal–Myeloid Features

**Figure 7:**
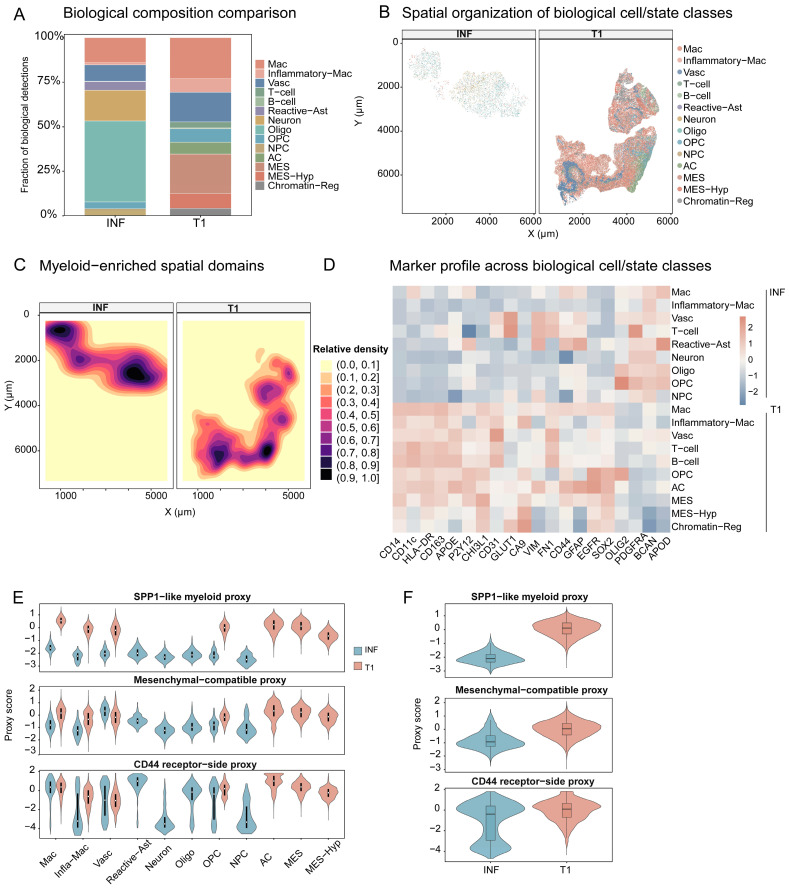
CODEX spatial protein imaging provides antibody-panel-based support for SPP1-associated mesenchymal–myeloid features. (**A**) Stacked bar plot showing the relative composition of annotated cell populations in the INF (infiltrative) and T1 (T1-enhancing tumor) regions. (**B**) Spatial scatter plots showing the distribution of annotated cell types at their original tissue coordinates in INF and T1. (**C**) Two-dimensional kernel density maps of myeloid cells highlighting myeloid-enriched spatial domains in each region. (**D**) Heatmap showing scaled protein expression of selected markers across annotated cell populations and regions. (**E**) Cell-type-resolved distributions of the SPP1-like myeloid proxy, mesenchymal-compatible proxy, and CD44 receptor-side proxy. Proxy scores were calculated as marker-based composite scores from normalized expression values of predefined antibody markers representing the corresponding protein feature sets. (**F**) Regional comparison of the same three proxy scores between INF and T1. Because these proxy scores were derived from a limited antibody panel, they should be interpreted as surrogate protein-level contextual readouts rather than direct measurements of the full *SPP1*-associated transcriptional program or SPP1-mediated ligand–receptor activity.

To extend our transcriptomic and spatial transcriptomic findings with an orthogonal spatial protein-level readout, we analyzed CODEX data from the INF and T1 regions of the ZH916 sample (Figure 7). Because the CODEX panel provides antibody-channel measurements rather than direct measurement of the full *SPP1*-associated transcriptional program or *SPP1*-mediated ligand–receptor activity, these analyses were interpreted as surrogate protein-level contextual support. The two regions showed distinct cellular compositions: INF displayed a more uneven distribution dominated by a limited subset of annotated populations, whereas T1 exhibited a broader and more heterogeneous cellular composition (Figure 7A). Mapping these cells back to their original tissue coordinates showed that the INF field comprised more spatially separated segments, whereas the T1 field spanned a larger and more continuous region with intermixing of multiple cell populations (Figure 7B).

We next examined the spatial distribution of myeloid cells. Two-dimensional density mapping showed myeloid-enriched spatial domains in both INF and T1, indicating regionally concentrated rather than uniform myeloid distributions (Figure 7C). In INF, these domains appeared more segmented and distributed across separated tissue areas, whereas in T1, high-density myeloid regions were concentrated within a more continuous tissue structure. Consistent with these architectural differences, the heatmap of marker expression showed region- and cell-type-associated protein variation across the two areas (Figure 7D), indicating broader regional differences in the local protein landscape beyond cell composition alone.

To relate these protein-level patterns to the SPP1-associated mesenchymal–myeloid framework, we calculated three marker-based composite scores: an SPP1-like myeloid proxy, a mesenchymal-compatible proxy, and a CD44 receptor-side proxy. Across annotated cell populations, the SPP1-like myeloid proxy was concentrated in a restricted subset of cell populations, consistent with selective enrichment of SPP1-associated myeloid features, whereas the mesenchymal-compatible proxy and CD44 receptor-side proxy showed broader but structured distributions across cell types (Figure 7E). At the regional level, all three proxy scores were generally higher in T1 than in INF (Figure 7F).

### 3.9. Clinical Associations of SPP1 Expression and an SPP1-Associated Mesenchymal Signature

**Figure 8:**
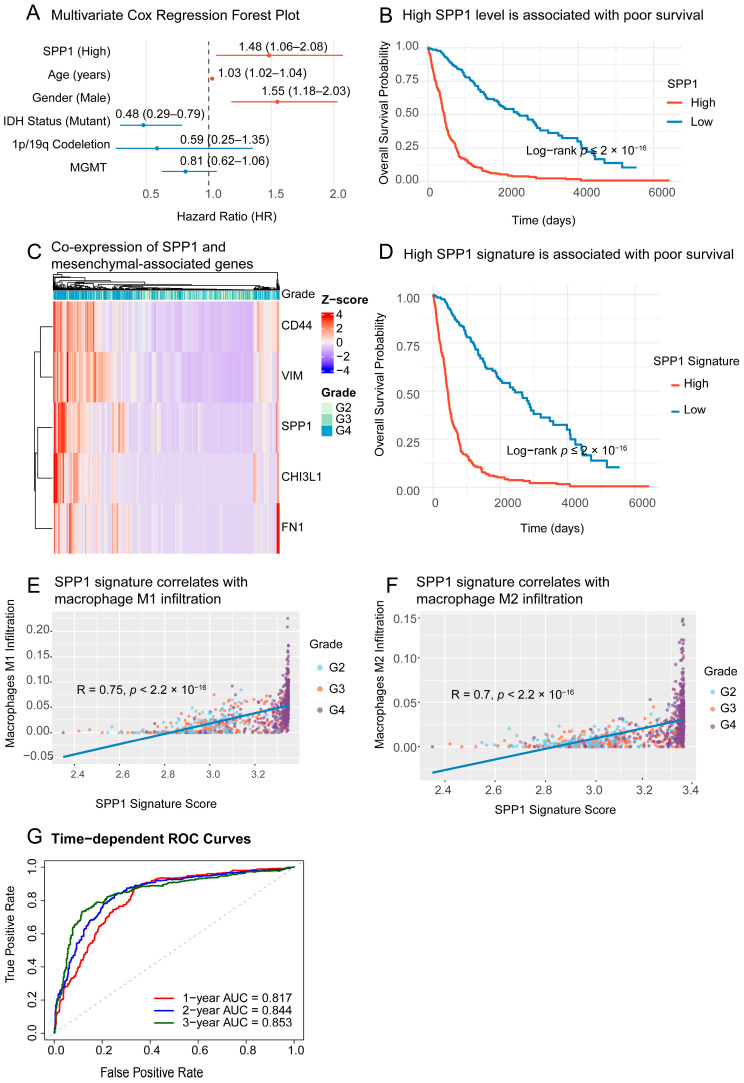
*SPP1* expression and an *SPP1*-associated mesenchymal signature are associated with poorer overall survival in GBM and macrophage-associated signatures across glioma cohorts. (**A**) Multivariable Cox regression analysis of overall survival in TCGA-GBM. Forest plot showing hazard ratios (HRs) and 95% confidence intervals (CIs) from a Cox proportional hazards model including *SPP1* expression, age, sex, IDH status, 1p/19q codeletion status, and MGMT status. High *SPP1* expression was associated with poorer overall survival after adjustment for these covariates (HR = 1.48, 95% CI: 1.06–2.08). (**B**) Kaplan–Meier survival curves showing overall survival probability stratified by *SPP1* expression level in TCGA-GBM. The high-expression group showed shorter overall survival than the low-expression group (log-rank *p* ≤ 2 × 10^−16^). Time is measured in days from diagnosis to death or last follow-up. (**C**) Co-expression of *SPP1* and mesenchymal-associated genes in the combined TCGA glioma cohort. Heatmap showing z-score-normalized expression levels of *SPP1* and key mesenchymal markers, including *CD44*, *VIM*, *CHI3L1*, and *FN1*, across glioma samples ordered by *SPP1* expression level. Samples are stratified by WHO tumor grade, as indicated by the color bar. (**D**) Kaplan–Meier survival curves showing overall survival probability stratified by the *SPP1*-associated mesenchymal signature score in TCGA-GBM. The high-score group showed shorter overall survival than the low-score group (log-rank *p* ≤ 2 × 10^−16^). (**E**,**F**) Associations between the *SPP1*-associated mesenchymal signature score and macrophage-associated xCell signatures in the combined TCGA glioma cohort. Scatter plots show correlations between the *SPP1*-associated mesenchymal signature score and estimated M1-like (**E**) and M2-like (**F**) macrophage-associated signatures. Each point represents a tumor sample, colored by histological grade. Positive associations were observed for both signatures using Spearman correlation. (**G**) Time-dependent receiver operating characteristic curves showing internal cohort discriminative performance for overall survival in TCGA-GBM based on the *SPP1*-associated mesenchymal signature score. The area under the curve values at 1, 2, and 3 years were 0.817, 0.844, and 0.853, respectively.

To assess the clinical relevance of *SPP1* expression and related mesenchymal transcriptional programs, we analyzed bulk transcriptomic and survival data from TCGA cohorts. The clinical analysis included 423 records from 323 unique patients. The multivariable Cox regression analysis was performed on 289 records from 240 unique patients with complete overall survival information, group assignment, and available covariates, including age, sex, IDH status, 1p/19q codeletion status, and MGMT status (Supplementary Table S5). Within the TCGA-GBM cohort, elevated *SPP1* expression was significantly associated with poorer overall survival in a multivariable Cox regression model adjusted for age, sex, IDH status, 1p/19q codeletion status, and MGMT status (HR = 1.48, 95% CI 1.06–2.08; Figure 8A). Kaplan–Meier analysis similarly showed significantly shorter survival among patients with *SPP1*-high tumors (log-rank *p* ≤ 2 × 10^−16^; Figure 8B).

To further evaluate *SPP1*-associated mesenchymal features at the molecular level, we examined the combined TCGA glioma cohort. *SPP1* showed coordinated expression with canonical mesenchymal markers, including *CD44*, *VIM*, *CHI3L1*, and *FN1*, forming a coherent *SPP1*-associated mesenchymal feature set (Figure 8C). To quantify this coordinated expression pattern at the tumor level, we defined an *SPP1*-associated mesenchymal signature consisting of *SPP1*, *CD44*, *VIM*, *CHI3L1*, and *FN1* and calculated sample-level activity scores. Patients with higher *SPP1*-associated mesenchymal signature scores had significantly shorter overall survival in the TCGA-GBM cohort (log-rank *p* ≤ 2 × 10^−16^; Figure 8D). Time-dependent receiver operating characteristic analysis provided an internal assessment of discriminative performance for overall survival at 1-, 2-, and 3-year time points in TCGA-GBM (AUC = 0.817, 0.844, and 0.853, respectively; Figure 8G).

Because macrophage-related transcriptional signatures vary across glioma grades [3,29], and because analyses in the combined TCGA glioma cohort may partly reflect underlying grade structure, we next examined the association between the *SPP1*-associated mesenchymal signature and xCell-estimated macrophage-associated signatures in this combined cohort. The *SPP1*-associated mesenchymal signature score was strongly correlated with both M1-like and M2-like macrophage-associated signatures (rho = 0.75, q = 2.89 × 10^−156^; rho = 0.70, q = 1.98 × 10^−127^, respectively; Figure 8E,F). Similar correlations were retained in GBM samples (rho = 0.627 for M1-like and rho = 0.470 for M2-like signatures) (Supplementary Table S6). These results support a transcriptional association between *SPP1*-related mesenchymal activity and myeloid-rich tumor microenvironments across glioma cohorts.

## 4. Discussion

A central message of this study is that *SPP1*-associated biology in GBM may be better understood in spatial and microenvironmental terms rather than as a diffuse inflammatory signal alone. Across single-cell, spatial transcriptomic, computational, tissue-context, spatial protein-level, and clinical association analyses, our findings support a model in which *SPP1* is associated with mesenchymal-like tumor programs, inflammatory myeloid remodeling, and their local co-enrichment within restricted tumor regions. This framing shifts the interpretation of *SPP1* from a broadly expressed macrophage-associated marker toward a candidate readout of mesenchymal–myeloid organization in GBM.

### 4.1. SPP1 as a Readout of Coupled Mesenchymal-like and Myeloid Remodeling

One implication of these findings is that *SPP1* should be discussed in relation to coupled mesenchymal-like and myeloid remodeling rather than as an isolated macrophage marker. The MES-like state in GBM has long been linked to hypoxia, inflammatory signaling, macrophage-rich microenvironments, and treatment resistance [30,31], but recent work increasingly suggests that these features are embedded in broader microenvironment-driven programs rather than representing purely cell-intrinsic subtype labels [32,33]. Our data are consistent with this view at multiple levels: *SPP1* was concentrated in myeloid populations, preferentially enriched in MES-like malignant states, aligned with metabolically adaptive programs, and embedded in inflammatory regulatory modules rather than appearing as a generic inflammatory signal alone.

This interpretation is also consistent with recent work showing that glioma-associated myeloid programs are strongly shaped by local cues such as hypoxia, IL-1β, TGFβ, and treatment-related pressures, and that these programs cut across conventional lineage labels [34]. It further aligns with earlier and more recent studies implicating osteopontin in macrophage recruitment, adverse mesenchymal biology, and hypoxia-associated mesenchymal transition in glioma [8,35]. Taken together, these observations place *SPP1* at the intersection of mesenchymal-like plasticity and inflammatory myeloid remodeling.

### 4.2. From Co-Occurrence to Coordinated Mesenchymal–Myeloid Coupling

A second point is that the relationship among *SPP1*, MES-like malignant states, and myeloid activation is better interpreted as a context-dependent spatial organization than as uniform co-occurrence. This interpretation is supported by multiple complementary observations. CellChat analysis nominated *SPP1*–integrin and *SPP1*–*CD44* ligand–receptor co-expression probabilities involving malignant, myeloid, stromal, and immune compartments. In parallel, *SPP1* and *CD44* showed overlapping expression across malignant and myeloid subsets, *SPP1*-positive M2-like macrophage-associated cells were abundant in the myeloid compartment, and spatial analyses showed enrichment of myeloid, mesenchymal-associated, and ECM/angiogenic features around *SPP1*-high regions. Together, these findings support an *SPP1*-associated mesenchymal–myeloid framework, while section-level variation in MES-like enrichment suggests that this program is spatially heterogeneous rather than uniform across all GBM regions.

This interpretation is also consistent with the growing glioma literature. Glioma-specific studies have shown that macrophage-associated signaling can promote mesenchymal-like transitions, including *SPP1*/*CD44*-associated macrophage–tumor crosstalk [9], TREM1-associated TGFβ2/TGFβR signaling from tumor-associated macrophages to glioma stem cells [36], and hypoxia-induced osteopontin-positive glioma-associated macrophage programs linked to NF-κB-associated mesenchymal transition [35]. In that context, our CellChat and spatial co-abundance results are best viewed not as stand-alone mechanistic proof, but as hypothesis-generating support for a broader framework in which myeloid-associated programs may help shape malignant-state plasticity in GBM. Our data are consistent with placing *SPP1*-associated programs within this broader mesenchymal–myeloid interaction framework.

### 4.3. Spatial Restriction Is Central to the Biological Interpretation

A key contribution of the present study is its spatial framing of *SPP1*-associated biology. Our results suggest that *SPP1*-associated biology is not uniformly distributed across the tumor, but instead concentrates in restricted regions with estimated myeloid abundance, ECM/angiogenic programs, and elevated *SPP1*-related co-abundance, with MES-like signal varying by spatial section. This point matters because recent spatial studies increasingly argue that GBM is organized into biologically distinct regions and anatomical contexts rather than into a single continuous tumor ecology. Spatial multi-omics studies have shown that GBM cellular states are spatially segregated, locally paired, and shaped by inflammatory, metabolic, and hypoxic microenvironments [25,28]. More recent atlas-level and region-focused spatial transcriptomic work likewise supports the existence of anatomically distinct GBM regions with characteristic cellular and molecular patterns and region-specific vulnerabilities [37,38].

Our study extends this spatial framework by linking it specifically to *SPP1*-associated myeloid–ECM and mesenchymal–myeloid biology. Spatial transcriptomics supported local *SPP1*-high myeloid/ECM and mesenchymal-associated co-enrichment; computational perturbation analysis supported stress-responsive and mesenchymal/inflammatory network programs; HPA provided tissue-level protein context; Ivy GAP analysis associated related programs with core-like anatomic compartments; and CODEX offered antibody-panel-based spatial protein context for related myeloid and mesenchymal-compatible features. None of these layers alone establishes a functional compartment, but together they support the interpretation that *SPP1*-associated programs belong to a spatially restricted microenvironmental context. *SPP1* therefore appears informative not simply because it is expressed in relevant cell populations, but because it is associated with local tissue organization.

### 4.4. Implications for Current Models of GBM Plasticity and Myeloid Heterogeneity

These findings refine current models of GBM plasticity in two ways. First, they support the idea that MES-like states are best interpreted in relation to the local immune and stromal environment, rather than as purely intrinsic transcriptional endpoints. Second, they suggest that myeloid heterogeneity in glioma should be understood functionally and spatially, not only by lineage or marker-based subclassification. This aligns with recent work showing that glioma-associated myeloid programs can transcend conventional origin labels and are strongly shaped by local microenvironmental cues [34]. It also fits with spatially resolved models in which GBM cell states and microenvironmental programs are organized into distinct regional ecologies rather than being diffusely distributed across tumor tissue [25,28].

In this sense, our study is less about proposing a new subtype or proving a causal microenvironmental unit than about sharpening an organizational model. *SPP1*-associated programs connect three dimensions that are often discussed separately: malignant-state plasticity, inflammatory myeloid remodeling, and spatial tissue architecture. This may help connect tumor-state interpretation with microenvironmental organization and may explain why mesenchymal biology in GBM often appears both transcriptionally prominent and spatially uneven across tumors.

### 4.5. Clinical and Translational Implications

The clinical implications of these findings should be interpreted cautiously. In the TCGA-GBM cohort, elevated *SPP1* expression and a higher *SPP1*-associated mesenchymal signature score were associated with poorer overall survival, and elevated *SPP1* remained associated with worse outcome in a multivariable Cox model adjusted for major clinical and molecular covariates. These findings do not establish *SPP1* as a validated therapeutic target or prognostic biomarker, but they suggest that *SPP1*-related biology marks an adverse tumor context in which malignant plasticity and myeloid remodeling are coupled. That interpretation is compatible with prior GBM literature on osteopontin-mediated macrophage recruitment [8] and with newer work showing that myeloid states in glioma are functionally diverse and microenvironment-shaped rather than adequately captured by bulk abundance alone [34].

More broadly, the translational relevance of *SPP1* may lie not only in its expression level, but also in what it indicates about local tumor organization. If mesenchymal–myeloid biology is spatially restricted, then therapeutic strategies directed at inflammatory signaling, TAM programs, or *SPP1*-related ligand–receptor programs may need to be interpreted through region-specific biology rather than through bulk tumor averages alone. As spatial atlases of GBM continue to mature, region-aware biomarkers and more spatially informed therapeutic frameworks are likely to become more relevant [25,37].

### 4.6. Limitations

Several limitations should be acknowledged. First, this study is based on computational and observational analyses. Inferred ligand–receptor co-expression probabilities, CellChat communication outputs, spatial co-abundance scores, and scTenifoldKnk computational perturbation results should therefore be interpreted as hypothesis-generating network-level support rather than proof of causal *SPP1* signaling. Experimental perturbation in relevant model systems will be required to determine whether *SPP1* functions as a driver, stabilizer, or marker of the mesenchymal–myeloid state. Second, although the spatial analyses support an *SPP1*-associated mesenchymal–myeloid program, the relative contribution of myeloid, MES-like, and ECM/angiogenic features varied across sections. In addition, mixed-grade spatial transcriptomic datasets were used for contextual comparison, and GBM-specific interpretations were restricted to GBM samples and GBM-focused analyses wherever possible. CODEX analyses were also based on a limited antibody panel and should be interpreted as surrogate protein-level contextual support rather than direct measurement of the full *SPP1*-associated transcriptional program or *SPP1*-mediated ligand–receptor activity. Third, the clinical analyses were retrospective and internally assessed using public TCGA data. Although elevated *SPP1* expression and a higher *SPP1*-associated mesenchymal signature were associated with poorer overall survival in the TCGA-GBM cohort, these findings do not establish *SPP1* as a clinically validated prognostic biomarker. Larger GBM-focused cohorts, longitudinal sampling, and independent external validation will be needed to determine how *SPP1*-associated programs evolve during treatment or recurrence and to assess their clinical relevance. Despite these limitations, the convergence of single-cell, spatial transcriptomic, computational perturbation, tissue-context, spatial protein-level, and clinical association analyses supports an inferential *SPP1*-associated mesenchymal–myeloid framework in GBM.

## 5. Conclusions

In summary, our integrative multi-omics analyses support an inferential *SPP1*-associated spatial mesenchymal–myeloid program in GBM. By positioning *SPP1* at the intersection of malignant-state plasticity, inflammatory myeloid remodeling, and spatial tumor architecture, this study contributes to a more integrated view of how tumor-intrinsic and microenvironmental programs are associated in GBM. These findings nominate *SPP1* as a candidate readout of myeloid-rich and mesenchymal-associated spatial organization and provide a framework for future mechanistic, longitudinal, and translational studies.

## Data Availability

The data presented in this study are publicly available and were derived from public domain resources. Single-cell RNA-seq data are available in the Gene Expression Omnibus (GEO) under accession numbers GSE182109 and GSE174554 (https://www.ncbi.nlm.nih.gov/geo/, accessed on 13 February 2026). Spatial transcriptomic data are available in GEO under accession number GSE237183 (https://www.ncbi.nlm.nih.gov/geo/, accessed on 16 February 2026) and from the dataset reported by Ravi et al. (2022) [28], which is available in Dryad at https://doi.org/10.5061/dryad.h70rxwdmj, accessed on 16 February 2026. Ivy GAP data are available through the Ivy Glioblastoma Atlas Project portal (https://glioblastoma.alleninstitute.org/, accessed on 5 April 2026). Immunohistochemistry images are available through the Human Protein Atlas (https://www.proteinatlas.org/, accessed on 4 April 2026). Molecular and clinical data for TCGA-LGG and TCGA-GBM are available through the Genomic Data Commons (https://portal.gdc.cancer.gov/, accessed on 11 March 2026). The CODEX data analyzed in this study were derived from the ZH916 sample reported by Greenwald et al. (2024) [25] and are available in Zenodo as part of the glioma spatialomics dataset at https://doi.org/10.5281/zenodo.12624860, accessed on 5 April 2026. No new raw data were generated in this study. The analysis code and reproducibility scripts used in this study are available through the Open Science Framework at https://osf.io/qgtza/overview?view_only=61a29eb7466743a4a790f2544a3f471d, accessed on 10 April 2026.

## Supplementary Materials

The following supporting information can be downloaded at: https://www.mdpi.com/article/10.3390/genes17060610/s1, Figure S1. Robustness assessment of single-cell clustering across resolutions. Figure S2. Robustness analysis of *SPP1*-high spatial hotspot-associated programs across threshold definitions. Figure S3. Robustness of *SPP1*-associated local spatial enrichment across k-nearest-neighbor definitions. Table S1. PCA variance explained in the integrated single-cell RNA-seq analysis. Table S2. CellChat-inferred *SPP1* ligand–receptor source–target communication outputs. Table S3. Robustness analysis of *SPP1*-high spatial hotspot definitions across glioma sections. Table S4. Spatial k-nearest-neighbor robustness analysis of *SPP1*-associated local neighborhood features. Table S5. TCGA clinical annotation and covariate-complete survival analysis dataset. Table S6. Spearman correlations between *SPP1*-related features and xCell-estimated macrophage-associated signatures.

## Abbreviations

The following abbreviations are used in this manuscript:

GBM	glioblastoma
SPP1	secreted phosphoprotein 1
MES-like	mesenchymal-like
AC-like	astrocyte-like
OPC-like	oligodendrocyte progenitor-like
NPC-like	neural progenitor-like
TAM	tumor-associated macrophage
HPA	Human Protein Atlas
IHC	Immunohistochemistry
Ivy GAP	Ivy Glioblastoma Atlas Project
CODEX	CO-Detection by indEXing
TCGA	The Cancer Genome Atlas
LGG	lower-grade glioma
LE	Leading Edge
IT	Infiltrating Tumor
CT	Cellular Tumor
MP	Microvascular Proliferation
PCAN	Pseudopalisading Cells Around Necrosis
INF	infiltrative region
