# Supplementary material for: Integrative Multi-Omics Analysis Identifies an SPP1-Associated Spatial Mesenchymal–Myeloid Program in Glioblastoma
Source: Genes (Basel). 2026 May 28;17(6):610. doi: 10.3390/genes17060610 (PMC13298322; doi:10.3390/genes17060610)
Supplement: Supplementary file 1 [file genes-17-00610-s001.zip › Supplementary Figure.pdf]

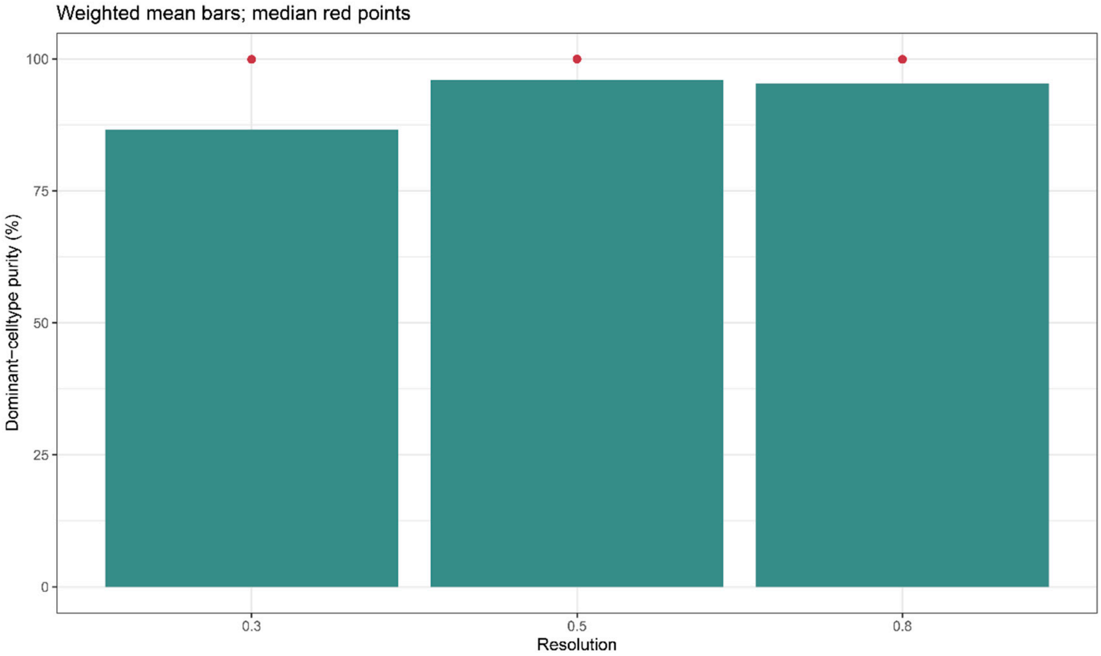

A

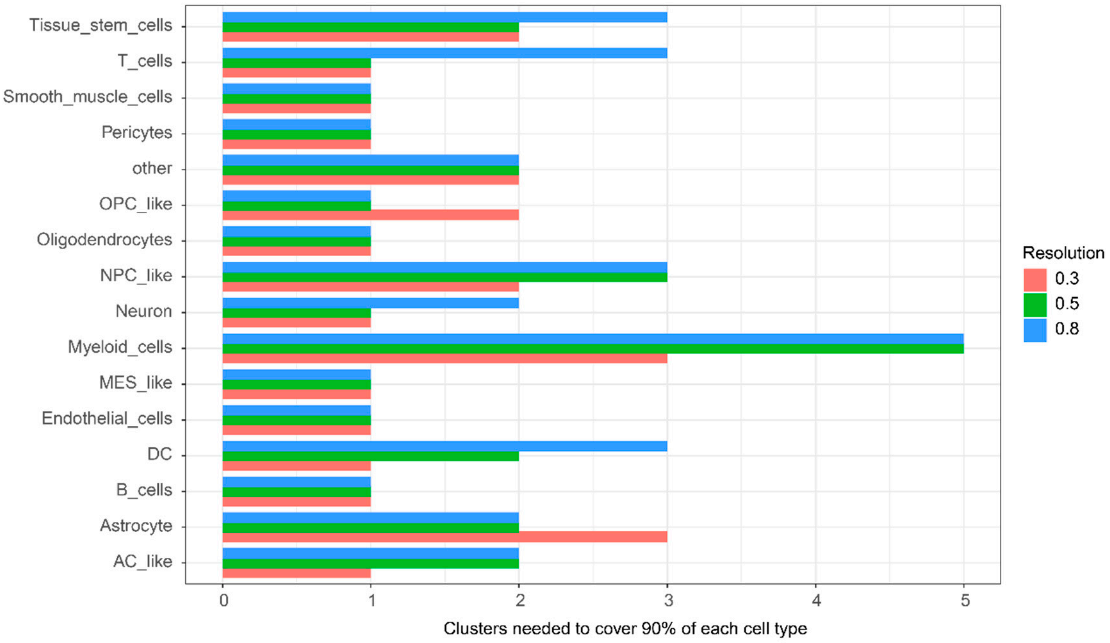

B

Supplementary Figure S1. Robustness assessment of single-cell clustering across resolutions. (A) Dominant-cell-type purity across Louvain clustering resolutions 0.3, 0.5, and 0.8. Bars represent the weighted mean dominant-cell-type purity for clusters generated at each resolution, and red points indicate the maximum cluster-level purity. **(B)** Dominant cell-type distribution across clustering resolutions. Bars indicate the number of clusters assigned to each dominant annotated cell type at resolutions 0.3, 0.5, and 0.8. The overall recovery of major malignant and non-malignant populations was preserved across resolutions, supporting the stability of the clustering strategy used in the single-cell analysis.

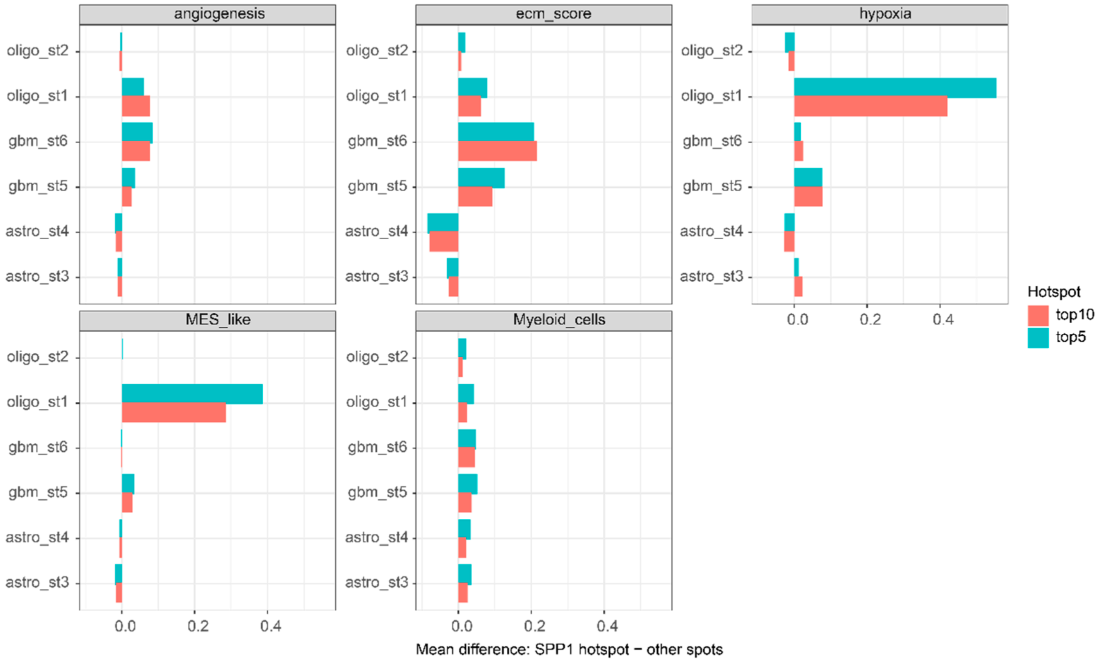

A

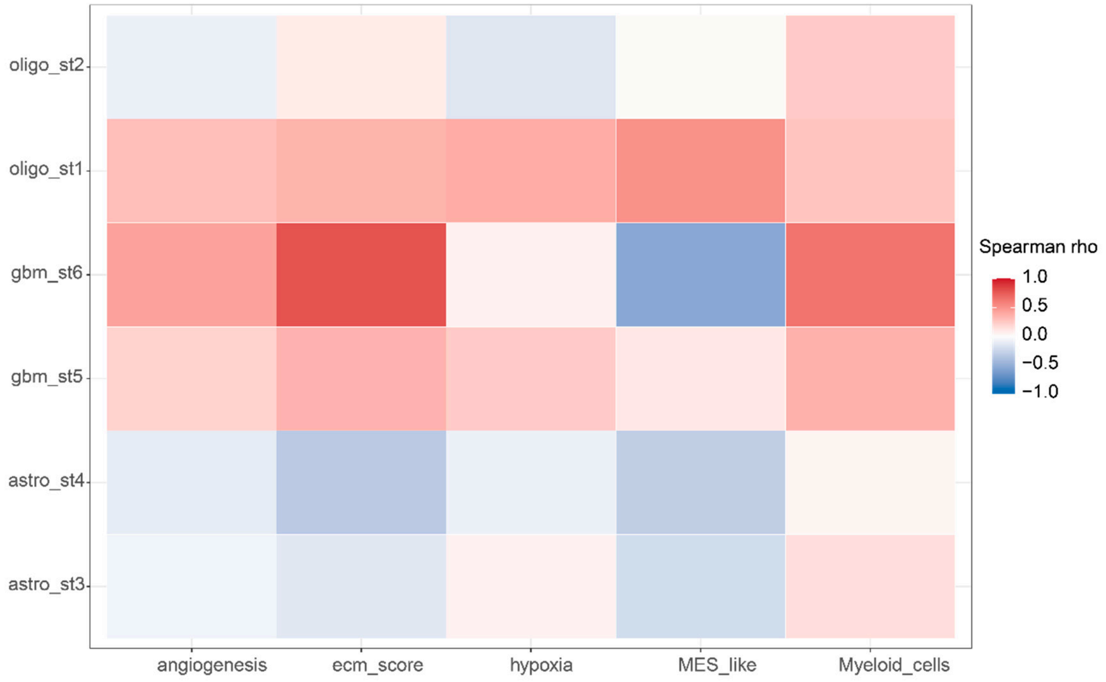

B

Supplementary Figure S2. Robustness analysis of *SPP1*-high spatial hotspot-associated programs across threshold definitions. (A) Bar plots summarizing hotspot-associated enrichment of angiogenesis, ECM, hypoxia, MES-like tumor signal, and estimated myeloid abundance across spatial transcriptomic sections. *SPP1*-high hotspots were defined using two thresholds, corresponding to the top 5% and top 10% of spots ranked by *SPP1* expression. For each feature and section, bars show the direction and magnitude of enrichment in *SPP1*-high hotspots relative to non-hotspot regions. **(B)** Heatmap summarizing the overall hotspot-associated enrichment pattern across spatial sections and biological programs. Red indicates positive enrichment in *SPP1*-high regions,

whereas blue indicates relative depletion. Across threshold definitions and samples, *SPP1*-high regions showed enrichment of myeloid-associated, mesenchymal-associated, and ECM/angiogenic features, supporting the robustness of the *SPP1*-associated spatial mesenchymal–myeloid program.

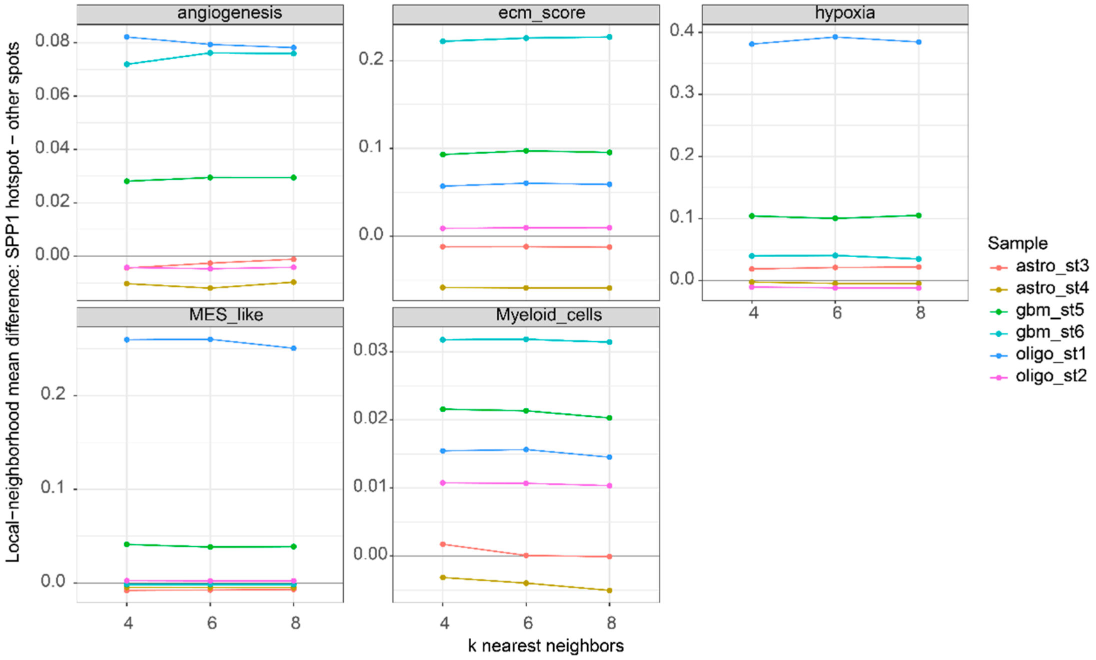

Supplementary Figure S3. Robustness of *SPP1*-associated local spatial enrichment across k-nearest-neighbor definitions. Local neighborhood analyses around *SPP1*-high regions were repeated using **k = 4, 6, and 8** nearest neighboring spots. Line plots show the enrichment or association strength of angiogenesis, ECM score, hypoxia, MES-like tumor signal, and estimated myeloid abundance across spatial transcriptomic sections. The largely preserved patterns across k values support the robustness of *SPP1*-associated local enrichment involving myeloid, mesenchymal-associated, and ECM/angiogenic features.
